# Mr. Davies, in Answer to Mr. Blair

**Published:** 1809-06

**Authors:** J. Davies


### To Mr. BLAIR.

Sir,

Ever accustomed to make truth the basis of my assertions, I come forward to substantiate what you think proper to term my "very harsh and personal reflections," with a degree of boldness which nothing short of such a conviction would inspire; it is however necessary to remark, that my few observations on the subject will be extremely brief; and as you request they may be explicit, I will likewise endeavour as much as possible, in this particular, to concede to your wishes. Having arranged your Inquiries under three distinct heads, it remains for me only to follow your own order; and in reply to the first, remark, that a little more attention to the observations by which you conceive yourself so unwarrantably censured, would have entirely superseded the necessity of urging the present Inquiry; since the remark to which you allude, was pointedly addressed to the "Deputation," and you, as constituting a part of it, consequently; though not solely implicated in it. This, however, as tending to confirm the charge, gives you a double claim to exact "the ground upon which I venture to insinuate such a charge of obliquity in your moral and medical character."

Any attempt to convince *you* of the justice of my observations on this head would naturally imply equal folly and inconsistency, since the same excessive prejudice, which prevents you from seeing those occasional proofs of the insecurity of vaccination, which are obvious to the eye of every impartial observer, would of course induce you you to believe, that no such principle actuates your conduct; for it would be an insult to common reason to suppose for a moment, that you would encourage prejudices which you *knew* to exist. This assertion, however, you may be inclined to doubt, unless I endeavour to explain what I meant to insinuate by being "*wilfully* deaf to the voice of conviction." I was not induced to adopt the phrase from even the remotest idea that, after the evidence of a clear, convincing, and well-authenticated instance of failure in vaccination, you would be guilty of so much obliquity as any longer to argue its infallibility; but, on the contrary, am thoroughly and unreservedly of opinion, that you do, in every respect, believe the practice as infallible as you have studiously represented it; and that your investigations of reported failures, prejudice alone has so prepossessed your mind in favour of the system, that the blame of insecurity, instead of attaching (as in some cases it undoubtedly ought to have done) to the inefficiency of its preventive influence, has been unexceptionably levelled against incomplete vaccination, improper management, the insertion of inactive virus, or, in fact, any occasional objection which might fortunately screen the favorite practice from public (and, in some instances, deserved) censure; any imputation, therefore, of obliquity was not, could not, have been intended; but an open, an unqualified charge of prejudice I did, and still continue to maintain.

Having gone through the two first, I now proceed to a very brief reply to your last inquiry, "Has he read all I have published on vaccination?" No! nor never shall! I have, Sir, read quite enough, both of your own and similar publications, to convince me that they flow from a source—infected, not with motives of "obliquity" either moral or professional, but with an obvious partiality of opinion; and surely, on no subject was there ever so much written to so little purpose as on the hacknied topic of vaccination. The wisest way, therefore, to guard the mind against the pernicious influence of prejudiced and uncandid sentiments, is, in my opinion, studiously to avoid the perusal of all publications whose known tendency is to inculcate any partiality of opinion. Actuated by this principle, I frankly confess I neither have, nor do I feel, any inclination whatever to learn the circumstances of the case which you seem so particularly to recommend to my attention, perfectly satisfied that the principle which gave rise to the animadversions by which you consider yourself aggrieved, is far, very far, from being exhausted.

I have, Sir, been induced to give you the above short, and, I think you will deem it, explicit explanation, in compliance with a very pointed request, which it would not have appeared liberal to evade; you must however excuse me when I assure you, that from a natural objection to any correspondence of a controversial nature, I am fully resolved on my part, to let the question rest here; for although I am perfectly convinced that I can satisfactorily substantiate whatever I have advanced, yet, as I have already put you in possession of all I have to say on the subject, any further correspondence would be not only unnecessary but superfluous; as inclination, therefore, and not cowardice, has induced me to recede from the field of contest, whether you accept this as a satisfactory explanation, or feel inclined to make any farther observations on the subject, will be a matter of the most perfect indifference to

Yours, &c.

